# Self-reported physical activity and lung function two months after cardiac surgery – a prospective cohort study

**DOI:** 10.1186/1749-8090-9-59

**Published:** 2014-03-28

**Authors:** Marcus Jonsson, Charlotte Urell, Margareta Emtner, Elisabeth Westerdahl

**Affiliations:** 1 Department of Physiotherapy, Örebro University Hospital, 701 85 Örebro, Sweden; 2 Department of Cardiothoracic surgery, Örebro University Hospital, 701 85 Örebro, Sweden; 3 Department of Neuroscience, Physiotherapy, Uppsala University, 751 24 Uppsala, Sweden; 4 Department of Medical Sciences, Respiratory Medicine and Allergology, Uppsala University, 751 85 Uppsala, Sweden; 5 School of Health and Medical Sciences, Örebro University, 701 82 Örebro, Sweden

**Keywords:** Cardiac surgery, Lung function, Physical activity

## Abstract

**Background:**

Physical activity has well-established positive health-related effects. Sedentary behaviour has been associated with postoperative complications and mortality after cardiac surgery. Patients undergoing cardiac surgery often suffer from impaired lung function postoperatively. The association between physical activity and lung function in cardiac surgery patients has not previously been reported.

**Methods:**

Patients undergoing cardiac surgery were followed up two months postoperatively. Physical activity was assessed on a four-category scale (sedentary, moderate activity, moderate regular exercise, and regular activity and exercise), modified from the Swedish National Institute of Public Health’s national survey. Formal lung function testing was performed preoperatively and two months postoperatively.

**Results:**

The sample included 283 patients (82% male). Two months after surgery, the level of physical activity had increased (p < 0.001) in the whole sample. Patients who remained active or increased their level of physical activity had significantly better recovery of lung function than patients who remained sedentary or had decreased their level of activity postoperatively in terms of vital capacity (94 ± 11% of preoperative value vs. 91 ± 9%; p = 0.03), inspiratory capacity (94 ± 14% vs. 88 ± 19%; p = 0.008), and total lung capacity (96 ± 11% vs. 90 ± 11%; p = 0.01).

**Conclusions:**

An increased level of physical activity, compared to preoperative level, was reported as early as two months after surgery. Our data shows that there could be a significant association between physical activity and recovery of lung function after cardiac surgery. The relationship between objectively measured physical activity and postoperative pulmonary recovery needs to be further examined to verify these results.

## Background

Physical activity has well-established health-related effects in both healthy and non-healthy individuals [1-4]. Recommendations on physical activity from the American Heart Association and the American College of Sports Medicine state that to promote and maintain health, all adults need moderate-intensity aerobic physical activity for a minimum of 30 minutes on five days each week, or vigorous-intensity aerobic activity for a minimum of 20 minutes on three days each week [5]. Combinations of moderate- and vigorous-intensity aerobic activity can be performed to meet these recommendations [5].

Population-based studies have shown that sedentary behaviour is associated with higher levels of cardio-metabolic and inflammatory biomarkers [6] and higher all-cause mortality [7]. Physical inactivity is associated with higher risk for cardiovascular disease [8]. Patients undergoing cardiac surgery have reduced lung function postoperatively, and often suffer from postoperative complications such as atelectasis and pleural effusion [9-12]. Patients who are sedentary preoperatively have longer hospital stay and a higher risk of postoperative pulmonary complications and mortality, compared to physically active patients [13,14].

It has been suggested that there is a positive association between level of physical activity and lung function in healthy adults [15], older individuals [16], and patients with chronic obstructive pulmonary disease [17,18]. However, the relationship between physical activity and lung function in patients undergoing cardiac surgery has not been studied.

The purpose of the present study was to describe pre- and postoperative self-reported physical activity and lung function in patients undergoing cardiac surgery. Our hypothesis was that sedentary patients have a more pronounced deterioration in lung function than more active patients.

## Methods

Patients were selected from a previous randomized controlled trial by Westerdahl et al. [19], including 313 patients undergoing cardiac surgery at Örebro University Hospital and Uppsala University Hospital, Sweden, between 2007 and 2011. The sample consisted of all patients who had answered a categorical question about physical activity (Table 1) both preoperatively and at two months after cardiac surgery (n = 283). Patients who had not answered the question postoperatively and were excluded (n = 30) did not differ compared to the remaining patients (n = 283), regarding age (p = 0.70), gender (p = 0.83), length of surgery (p = 0.27), or forced expiratory volume in one second (FEV1) (p = 0.62).

**Table 1:** A four-category scale for assessment of self-reported physical activity during the last month

Sedentary	You spend most of your time sedentary. You walk or are active in some other way for less than 2 hours per week.
Moderate activity	You walk or are active in some other way for at least 2 hours per week, usually without sweating.
Moderate regular exercise	You exercise regularly 1–2 times per week, at least 30 minutes per time, with activity that causes you to sweat.
Regular activity and exercise	You exercise regularly at least 3 times per week, at least 30 minutes per time, with activity that causes you to sweat.

The categorical question was answered preoperatively and two months after cardiac surgery.

All patients were scheduled for non-emergency coronary artery bypass grafting (CABG), valve surgery, or a combination of CABG and valve surgery. The patients underwent cardiac surgery under general anaesthesia, with or without cardiopulmonary bypass, using sternotomy as the surgical incision. All patients received the same pre- and postoperative care and treatment from nursing staff and physiotherapists in the cardiothoracic surgery ward during the hospital stay. This included general care, early mobilisation, analgesia, breathing exercises, and information about postoperative regimens.

Descriptive data (sex, age, weight, height, information on smoking, preoperative ejection fraction, and surgical data) were collected from medical records. Lung function and self-reported level of physical activity were assessed preoperatively and two months after surgery. Measurements of static and dynamic lung function were performed by experienced biomedical technicians at the Department of Physiology (Jaeger MasterScreen PFT/Bodybox, Intramedic AB, Bålsta, Sweden). The tests were performed according to the recommendations of the American Thoracic Society and the European Respiratory Society [20,21]. The patients performed the tests in a sitting position, wearing a nose clip. The highest value of three correctly performed tests was registered. Vital capacity (VC), forced vital capacity (FVC), forced expiratory volume in 1 second (FEV1), inspiratory capacity (IC), functional residual capacity (FRC), residual volume (RV), and total lung capacity (TLC) were assessed. Predicted values were related to age, gender, and height [22,23].

Self-reported leisure-time physical activity during the last month was estimated on a four-category scale (sedentary, moderate activity, moderate regular exercise, and regular activity and exercise; Table 1) modified from the Swedish National Institute of Public Health’s national survey [24]. Patients who were sedentary (category 1), or reported a lower postoperative level of activity in comparison with their preoperative level, were compared with patients remaining active (categories 2–4) or reporting a higher level.

Version 21.0 of the SPSS software package (SPSS Inc, Chicago, IL, USA) was used for the statistical analysis. Demographic data were analysed using descriptive statistics. Preoperative and postoperative values for self-reported leisure-time physical activity were compared using the Wilcoxon signed rank test, while values for patients who reported being sedentary or less active postoperatively than preoperatively, and patients who remained active or became more active were compared using the unpaired Student’s t-test or the Chi2-test. Postoperative lung function was expressed in terms of percentages of the preoperative values. The level of significance was set at p < 0.05. Written informed consent was obtained from each patient, and the study was approved by the Regional Ethical Review Board in Uppsala (ref 2007/160).

## Results

The sample consisted of 231 male (82%) and 52 female patients with a mean age of 67 ± 10 years. Patients underwent isolated CABG (n = 100), aortic and/or mitral valve repair (n = 135), or combination CABG and valve repair (n = 48). Before surgery, the lung function for the whole sample was within the normal range (VC: 91 ± 15% and FEV1: 90 ± 17% of predicted values). At the two-month follow-up, FEV1 was 93 ± 11% and FVC 94 ± 11% (p < 0.001) of preoperative values.

Preoperatively, 52 patients (18%) reported being sedentary (Table 2). They had significantly lower lung function than the more active patients (FVC: 84 ± 15% vs. 89 ± 15% of predicted values; p < 0.05). At the two-month follow-up, 23 patients (8%) reported being sedentary. In comparison to preoperative activity, 113 patients (40%) had a higher self-reported level of physical activity postoperatively (p < 0.001), 38 patients (13%) a lower level, and 132 patients (47%) the same level as before (Table 2).

**Table 2:** Distribution of individuals in the categories of physical activity, preoperatively and two months after surgery

**Level of physical activity**	**Preoperatively**	**Two months after surgery**
	**n (%)**	**n (%)**
Sedentary	52 (18.4)	23 (8.1)
Moderate activity	148 (52.3)	126 (44.5)
Moderate regular exercise	40 (14.1)	48 (17.0)
Regular activity and exercise	43 (15.2)	86 (30.4)

The level of physical activity increased postoperatively (p < 0.001).

**Figure 1:**
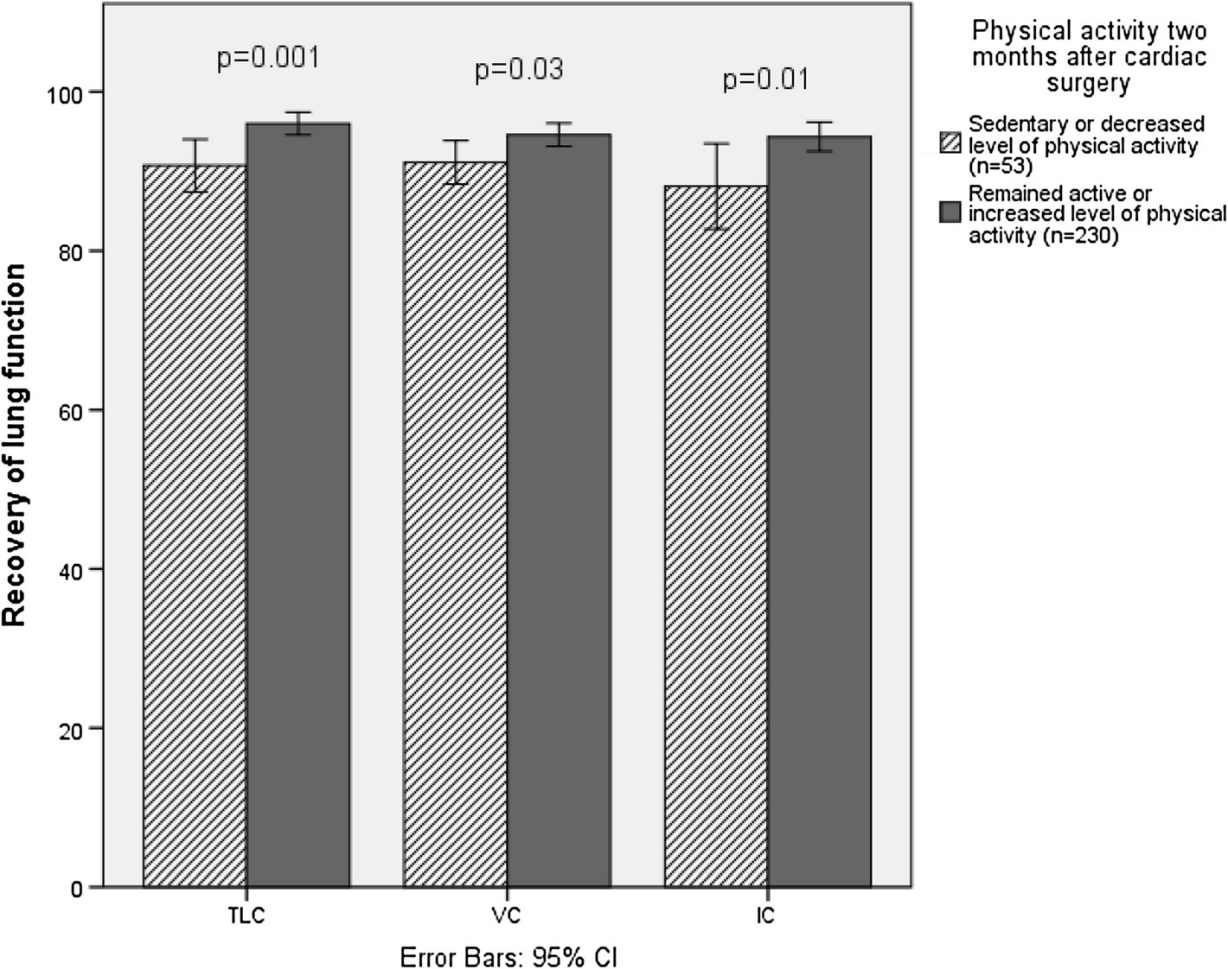
**Difference in recovery of lung function between sedentary and active patients.** Patients who remained active or increased their level of physical activity had better recovery of lung function (postoperative values expressed as percentages of preoperative values) than patients who remained sedentary or decreased their level of physical activity. TLC = total lung capacity, VC = vital capacity, IC = inspiratory capacity.

In total, 230 patients (81%) remained active or had become more active two months after surgery. These patients had significantly better recovery of lung function than the 53 patients who remained sedentary or reported a lower categorical level of physical activity two months after surgery, in terms of VC (94 ± 11% of preoperative value vs. 91 ± 9%; p = 0.03), TLC (96 ± 11% vs. 90 ± 11%; p = 0.01), and IC (94 ± 14% vs. 88 ± 19%: p = 0.008) (Table 3, Figure 1). There was no difference in preoperative lung function between the groups (Table 3). The groups were comparable in terms of age, smoking history, preoperative ejection fraction, and surgical variables (Table 4). Patients who remained sedentary or were less active postoperatively had significantly higher body mass index (BMI) than patients who remained active or became more active, as shown in Table 4.

**Table 3:** Difference in lung function between sedentary or less active and active patients

	**Before surgery**						**Two months after surgery**		**Difference between groups regarding change after 2 months (95% CI)**	
	**Sedentary/less active**	**Active**	**p-value**	**Sedentary/less active**	**Active**	**p-value**	**Sedentary/less active**	**Active**		**p-value**
**Variable**	**Value (L)**	**Value (L)**		**% predicted**	**% predicted**		**% preoperative**	**% preoperative**		
VC	3.9 ± 1	4.1 ± 1	0.22	88 ± 15	92 ± 15	0.10	91 ± 9	94 ± 11	3% (0.4 to 7)	0.027*
FVC	3.7 ± 1	3.9 ± 1	0.15	84 ± 15	89 ± 15	0.05	91 ± 10	94 ± 11	3% (-0.3 to 6)	0.076
FEV1	2.8 ± 1	2.9 ± 1	0.22	87 ± 17	90 ± 17	0.21	90 ± 11	93 ± 11	3% (-0.6 to 6)	0.119
IC	3.1 ± 1	3.1 ± 1	0.88	87 ± 20	93 ± 21	0.10	88 ± 19	94 ± 14	6% (2 to 11)	0.008*
FRC	3.3 ± 1	3.5 ± 1	0.19	109 ± 49	99 ± 28	0.06	96 ± 11	98 ± 12	2% (-2 to 6)	0.301
RV	2.6 ± 1	2.5 ± 1	0.64	108 ± 35	100 ± 25	0.05	95 ± 17	97 ± 14	2% (-2 to 7)	0.281
TLC	6.4 ± 1	6.6 ± 1	0.53	93 ± 12	93 ± 12	0.95	90 ± 11	96 ± 11	6% (2 to 9)	0.001*

Sedentary/less active = patients who remained sedentary or decreased their level of activity, two months after surgery. Active = patients who remained active or increased their level of activity two months after surgery.

Data are presented as mean ± SD (95% CI of the difference). The two months values are expressed as percentages of preoperative values. P-value refers to the difference between sedentary/less active (n = 53) and active patients (n = 230), *indicates significant differences (p < 0.05).

*VC* vital capacity, *FVC* forced vital capacity, *FEV1* forced expiratory volume in one second, *IC* inspiratory capacity, *FRC* functional residual capacity, *RV* residual volume, *TLC* total lung capacity.

**Table 4:** Descriptive data for the whole sample, and comparison of sedentary and active patients

**Variable**	**Whole sample (n = 283)**	**Sedentary or less active (n = 53)**	**Active (n = 230)**	**P-value**
Sex (male/female)	231/52	39/14	192/38	0.09
Age (years)	67 ± 10	67 ± 9	67 ± 10	0.8
BMI (kg/m^2^)	27 ± 4.3	28.5 ± 5	26.8 ± 4	0.01*
Never smoked/ex-smoker/smoker (%)	44/51/5	38/58/4	46/50/4	0.5
EF (%)	54 ± 9	54 ± 9	54 ± 9	0.99
Length of anaesthesia (hours)	10 ± 4	10 ± 3	10 ± 4	0.15
Aortic occlusion (minutes)	79 ± 40	78 ± 41	79 ± 40	0.79
ECC (minutes)	112 ± 50	108 ± 47	113 ± 50	0.52
Length of surgery (hours)	4 ± 1	4 ± 1	4 ± 1	0.97

Sedentary or less active = patients who remained sedentary or decreased their level of activity, two months after surgery. Active = patients who remained active or increased their level of activity. *BMI* body mass index, *EF* preoperative ejection fraction, *ECC* extracorporeal circulation. P-value refers to the difference between sedentary and active patients, *indicates significant differences (p < 0.05).

## Discussion

In this prospective cohort study, patients who reported being sedentary preoperatively had lower lung function than more active patients. Patients who remained active or increased their level of physical activity two months after surgery had better recovery of lung function than patients who remained sedentary or reported a lower level of physical activity two months postoperatively. To our knowledge, this is the first study investigating self-reported physical activity and lung function in patients undergoing cardiac surgery. The clinical significance of these results is difficult to interpret. However, even a small postoperative decrease in lung function might be clinically important to patients with high age or pulmonary disease. Besides the well-established positive cardiovascular effects of physical activity, this result indicates a beneficial effect on pulmonary recovery after cardiac surgery.

The patients in our study who remained sedentary or reported a lower level of activity postoperatively had significantly higher BMI than the patients who remained active or increased their level of activity. A high BMI has been associated with postoperative atrial fibrillation [25], superficial infection [26], deep sternal infection [27], and readmission to the intensive care unit [28] after cardiac surgery. The BMI in both groups in our study was below the risk levels reported in these studies. It is not clear if sedentary behaviour is associated with these postoperative complications, but the relationship with higher BMI calls for more research in the area.

The patients in our study reported a higher level of physical activity, as early as two months after surgery; that is, more patients were active after surgery than before. A few studies have investigated physical activity after CABG, with somewhat contradictory results [13,29-31]. In accordance with our data, Markou et al. [30] and Nery et al. [13] reported significantly increased physical activity for the study population one year after CABG, while Nery et al. [31] showed an increase in the number of physically active patients two years after CABG. In contrast, Barandon et al. [29] showed a decrease in physical activity, compared with preoperatively, seven months after CABG. The fact that our sample of patients reported being more physically active postoperatively might be an indication of the positive effect of the cardiac surgery on functional status; that is, the patients had an improved ability to be physically active. This could lead to improved quality of life, a lower incidence of cardiac events, and reduced mortality.

Our sample showed decreased lung function two months after cardiac surgery, in comparison to preoperative values. This impact of cardiac surgery on lung function is in accordance with earlier results [9-12]. Patients who categorized themselves as being sedentary preoperatively had lower lung function, expressed as percent of predicted, than their more active counterparts. This is consistent with the results reported by Sieverdes et al. [32], who studied healthy men. The additional contribution of our results is the fact that the patients who increased or maintained their level of activity had significantly better recovery of lung function than patients who remained sedentary or reduced their level of activity.

Impaired lung function could lead to postoperative pulmonary complications, and has been shown to have an impact on mortality and quality of life [18,33]. These findings point to the importance of preventing or minimizing the impairment of pulmonary function after cardiac surgery. According to our results, a reduction in sedentary time could be one way to achieve this. Sedentary behaviour has been associated with postoperative complications, increased levels of cardio-metabolic and inflammatory biomarkers, and mortality [6,7]. Our results are the first findings indicating that sedentary patients have reduced recovery of lung function after cardiac surgery. This further emphasizes the importance of reducing sedentary behaviour in patients undergoing cardiac surgery.

A limitation of our study is the use of a self-reported physical activity questionnaire; this is not as exact as the use of more objective methods such as accelerometers or pedometers, and hence could be a source of bias. The categorical question used in our study has not been formally validated for cardiac surgery patients. Another limitation is the relatively small size of the study, and the disparity in size between the patient groups. Despite this, we found significant differences in recovery of lung function.

The relationship between postoperative pulmonary recovery and objectively measured physical activity should be further examined, in order to find the minimum level of physical activity needed to reach a clinically relevant improvement in lung function.

## Conclusions

An increased level of physical activity, compared to preoperative level, was reported as early as two months after surgery. Our data shows that there could be a significant association between physical activity and recovery of lung function after cardiac surgery. The relationship between objectively measured physical activity and postoperative pulmonary recovery needs to be further examined to verify these results.

## Abbreviations

BMI: Body mass index; CABG: Coronary artery bypass grafting; FEV1: Forced expiratory volume in one second; FRC: Functional residual capacity; FVC: Forced vital capacity; IC: Inspiratory capacity; RV: Residual volume; TLC: Total lung capacity; VC: Vital capacity.

## Competing interests

The authors declare no competing interests.

## Authors’ contributions

MJ participated in the design of the study, performed data collection, performed the statistical analysis, and wrote the manuscript. CU participated in the design of the study, performed data collection, and helped to draft the final manuscript. ME participated in the design of the study and helped to draft the final manuscript. EW participated in the design of the study and the writing of the manuscript. All authors read and approved the final manuscript.
